# Supplementary material for: An implantable system to restore hemodynamic stability after spinal cord injury
Source: Nat Med. 2025 Sep 17;31(9):2946–57. doi: 10.1038/s41591-025-03614-w (PMC12443590; doi:10.1038/s41591-025-03614-w)
Supplement: Supplementary file 1 — Supplementary Notes 1 and 2, Supplementary Figs. 1 and 2 and Supplementary Tables 1–8. [file 41591_2025_3614_MOESM1_ESM.pdf]

nature medicine

Supplementary information https://doi.org/10.1038/s41591-025-03614-w

An implantable system to restore hemodynamic stability after spinal cord injury

In the format provided by the authors and unedited

Supplementary Note 1: Extended epidemiological results related to orthostatic hypotension in people with SCI

EES is a promising therapy to address the lack of satisfying management options for hemodynamic instability due to SCI. However, EES requires a neurosurgical intervention that must be weighed against the risks and benefits of the procedure. Establishing this balance requires an understanding of the prevalence, symptomatology, and effectiveness of current management strategies. While hypotension is a recognized medically refractory complication of SCI[2], these epidemiological factors have not been quantified conclusively.

To address this knowledge gap, we analyzed the Spinal Cord Injury Community Survey[26,27] (SCICS), which includes self-reported information on symptoms of orthostatic hypotension and demographic information in 1,479 individuals living with chronic SCI **(Supplementary Data Fig. 1a)**. This analysis revealed that 78% of individuals with tetraplegia had been told by a medical practitioner that they have orthostatic hypotension. Of this subset of individuals who were diagnosed, 28% of them were being treated for orthostatic hypotension, yet 91% still experienced symptoms **(Fig. 1a)**. Orthostatic hypotension was still present in half the people with paraplegia **(Supplementary Data Fig. 1b)**, and analysis of only individuals with complete (ASIA A) injuries led to comparable results **(Supplementary Data Fig. 1c)**. Together these results suggested that conservative treatments for orthostatic hypotension are insufficient to manage the ongoing symptomatology.

This analysis compelled us to characterize the pattern of hypotension-related symptoms due to SCI. For this purpose, we surveyed a diverse sample of 254 individuals with SCI from 26 different countries, which included responses to the Autonomic Dysfunction Following Spinal Cord Injury (ADFSCI) rating scale. When considering individuals with tetraplegia, we found that every individual who responded to the survey experienced symptoms of hypotension throughout the day. These symptoms were primarily characterized by lightheadedness, dizziness, fatigue, blurred vision, and weakness throughout the day (**Fig. 1b**). As expected, the frequency and severity of hypotension-related symptoms were higher in people with tetraplegia compared to people with paraplegia, and in those with complete SCI compared to those with incomplete SCI (**Supplementary Data Fig. 1e**).

To link these outcomes to objective physiological quantifications, we conducted a formal 10-minute tilt table test in a subset of these individuals (**Fig. 1c, Supplementary Data Fig. 2a-d** and **Supplementary Data Table 3**). We found that individuals who met the criteria for orthostatic hypotension during the tilt-table test reported significantly more hypotensive symptoms (**Fig. 1d-f** and **Supplementary Data Fig. 2e-i**).

These results established that i) the vast majority of individuals living with tetraplegia experience severe and persistent symptoms of hypotension; ii) hypotension is accompanied by quantifiable symptomatology that increases with the level and completeness of injury, iii) the ADFSCI is a valid self-reported measure of hypotension, and iv) the management of hypotension is insufficient to reduce the symptomatology.

We concluded that these outcomes, combined with the long-term consequences of medically refractory hypotension, justified the consideration of therapeutic strategies involving surgical interventions to manage hypotension due to SCI.

Supplementary Note 2: Requirements for a system to improve hemodynamic instability after SCI

Having identified the optimal location over which EES must be delivered to regulate blood pressure, we next sought to develop an implanted system that leveraged this understanding with the goal of establishing a therapy to improve the management of hemodynamic instability in people with SCI. We reasoned that such a system must combine the following features.

First, the implantable neurostimulation platform should include the capacity to adjust EES in closed-loop. Indeed, while continuous EES is sufficient to stabilize blood pressure in static conditions, stabilization of blood pressure during dynamic changes in body orientation requires constant adjustment of EES amplitudes[1]. Moreover, constant adjustment of EES would be ideal in acute care settings wherein blood pressure lability impacts the neurological recovery of patients[5–8].

Second, the paddle lead must include an optimal configuration of electrodes to target the ensemble of dorsal root entry zones innervating the hemodynamic hotspot, while avoiding the undesired recruitment of sensory axons ascending in the dorsal columns. Since functional and anatomical experiments in mice, rats, nonhuman primates, and six humans revealed that the correct location to deliver EES is centered over the last three thoracic segments of the spinal cord, we concluded that the paddle lead must cover the entire extent of this hemodynamic hotspot.

Third, the hardware and software components of the system must incorporate practical interfaces to support the rapid configuration of EES by physicians, and enable patients to operate the therapy safely and independently despite accessibility limitations.

We aimed to develop a system that fulfilled these requirements and to establish the safety and efficacy of each component embedded in this new purpose-built implantable system through a series of stepwise clinical validations.

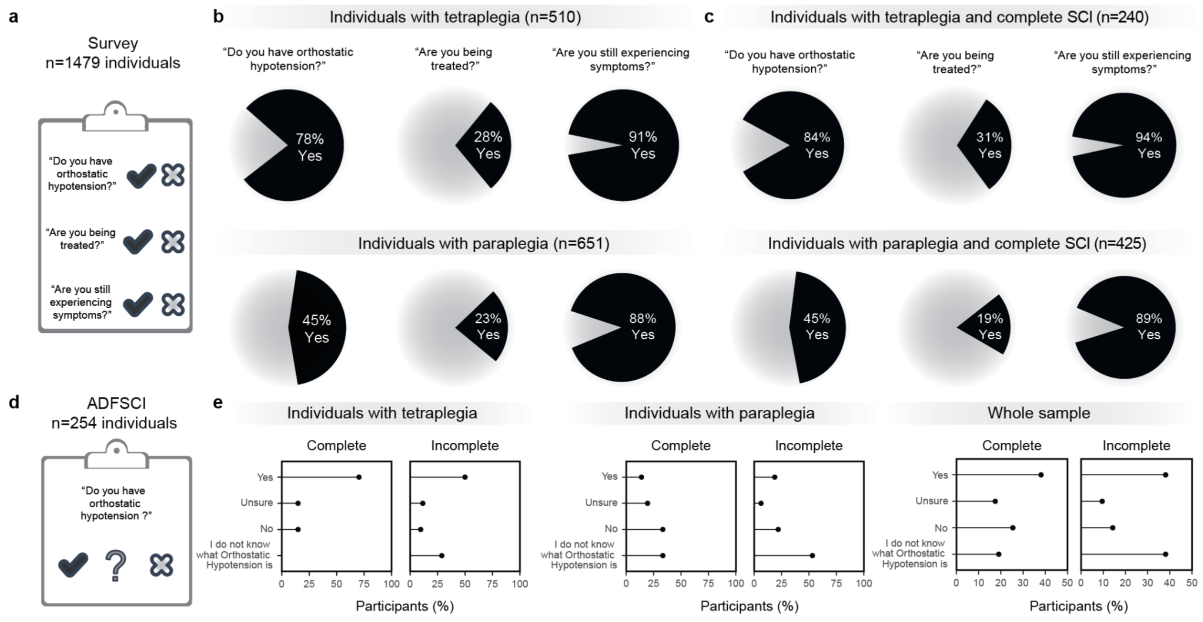

Supplementary Data Fig. 1 | Orthostatic hypotension is a medically refractory condition in people with SCI

a, Survey on the presence of orthostatic hypotension and efficacy of its management conducted in a cohort of 1479 individuals with SCI as part of the Spinal Cord Injury Community Survey (SCICS).

b, Prevalence of orthostatic hypotension and efficacy of its management quantified from the SCICS survey (n = 1161 of 1479 valid responses, of which 510 individuals with tetraplegia and 651 with paraplegia).

c, Prevalence of orthostatic hypotension and efficacy of its management in individuals with functionally complete SCI quantified from the SCICS survey (n = 665 individuals with complete SCI, of which n= 240 individuals with tetraplegia and n= 425 with paraplegia).

d, Survey on the presence of orthostatic hypotension conducted in 254 individuals with SCI, acquired with the Autonomic Dysfunction Following Spinal Cord Injury questionnaire (ADFSCI).

e, Percentage of individuals who report orthostatic hypotension, segregated by the severity of the SCI in people with tetraplegia (n = 79), paraplegia (n = 68), and for the entire sample (n = 147).

See **Supplementary Data 1** for source data and statistics.

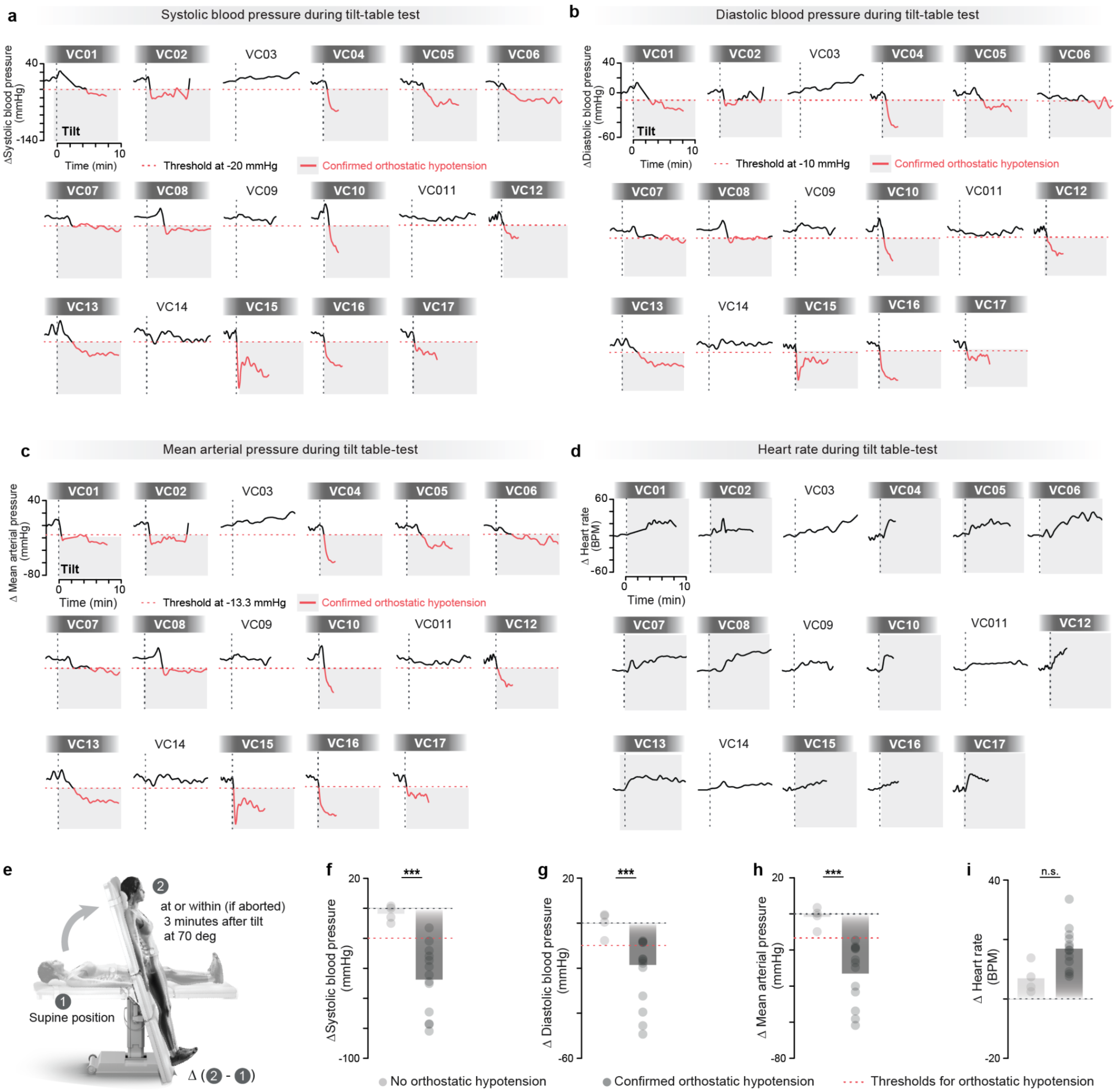

a Systolic blood pressure during tilt-table test
b Diastolic blood pressure during tilt-table test
ΔSystolic blood pressure (mmHg)
ΔDiastolic blood pressure (mmHg)
Tilt
Time (min)
Threshold at -20 mmHg
Threshold at -10 mmHg
Confirmed orthostatic hypotension
VC01 VC02 VC03 VC04 VC05 VC06 VC07 VC08 VC09 VC10 VC011 VC12 VC13 VC14 VC15 VC16 VC17
c Mean arterial pressure during tilt table-test
d Heart rate during tilt table-test
Δ Mean arterial pressure (mmHg)
Δ Heart rate (BPM)
Threshold at -13.3 mmHg
e
at or within (if aborted) 3 minutes after tilt at 70 deg
Supine position
Δ (2 - 1)
f ΔSystolic blood pressure (mmHg)
g Δ Diastolic blood pressure (mmHg)
h Δ Mean arterial pressure (mmHg)
i Δ Heart rate (BPM)
***
n.s.
No orthostatic hypotension
Confirmed orthostatic hypotension
Thresholds for orthostatic hypotension

Supplementary Data Fig. 2 | Validation of ADFSCI survey in a random cohort of individuals with SCI

a, Change in systolic blood pressure in response to an orthostatic challenge from a random sample of individuals who had filled out the Autonomic Dysfunction Following Spinal Cord Injury. The threshold crossing for confirmed orthostatic hypotension (red) was based on criteria established by the American Autonomic Society and the American Academy of Neurology [46].

b, As in **a,** for diastolic blood pressure.

c, As in **a,** for mean arterial pressure.

d, As in **a,** for heart rate.

e, Scheme of the tilt table test described in **a-d**.

f, Average change in systolic blood pressure within 3 minutes of a 10-minute tilt-table test (n = 17, independent samples two-tailed t-test; t-value = 6.44, p = 1.19e-05). Each dot reports quantifications from a single participant who did not present with orthostatic hypotension (light grey) or who presented confirmed orthostatic hypotension (dark grey).

g, Same as in **f,** for diastolic blood pressure (independent samples two-tailed t-test; t = 4.51; p = 4.78e-04).

h, Same as in **f**, for mean arterial pressure (independent samples two-tailed t-test; t = 5.78; p = 4.27e-05).

i, Same as in **f**, for heart rate (independent samples two-tailed t-test; t = -3.13; p = 1.60e-02). See **Supplementary Data 1** for source data and statistics.

Participant	Age at assessment (y)	Sex	Neurological Level of Injury	American Spinal Injury Association Impairment Scale (ASIA)	Time after injury at assessment (y)
VC01	49	M	C7/C8	A	10
VC02	42	F	T4	B/C	25
VC03	66	M	C3-C6	A	5
VC04	43	F	Low cervical	C	23
VC05	65	M	C4	A	11
VC06	33	F	Low cervical	C	11
VC07	49	F	Low cervical	A	26
VC08	42	F	T6	A	8
VC09	23	M	C4	A	5
VC10	24	M	C6/C7	A	2
VC11	46	F	C7	A	3.5
VC12	41	M	C4	A	21
VC13	39	F	C4	B	14
VC14	30	M	Low cervical	A	1
VC15	37	M	C4	B	0.8
VC16	42	M	Low cervical	B	1.5
VC17	66	M	High cervical	B	2.5

Supplementary Data Table 1 | Validation cohort demographics
Participant demographics for validation cohort.

	**Trial 1**<br>NCT04994886			**Trial 2**<br>NCT05044923			**Trial 3**<br>NCT05111093							**Trial 4**<br>NCT05941819
**Participants**	P1	P2	P3	P4	P5	P6	P7	P8	P9	P10	P11	P12	P13	P14
**Investigator Site**	CHUV	CHUV	CHUV	UFC	UFC	UFC	CHUV	CHUV	CHUV	CHUV	CHUV	CHUV	CHUV	Sint Martenskliniek
**City**	Lausanne	Lausanne	Lausanne	Calgary	Calgary	Calgary	Lausanne	Lausanne	Lausanne	Lausanne	Lausanne	Lausanne	Lausanne	Nijmegen
**Sex**	M	M	M	M	F	M	M	F	F	M	F	F	M	M
**Age at study enrolment (y)**	34	30	30	24	39	41	42	23	33	73	36	64	29	31
**Date of surgery**	30-Jun-21	11-Nov-21	1-Dec-22	25-Aug-22	27-Sep-22	14-Jun-23	9-May-22	31-Aug-22	26-Sep-22	21-Nov-22	15-May-23	22-May-23	4-Dec-23	29-Sep-23
**Time after injury at enrolment**	8 y	9 y	2 y	3 y	2 y	22 y	7 m	9 m	6 y	45 y	2 y	3 y	1 y	4 y
**American Spinal Injury Association Impairment Scale (ASIA)**	A	A	A	A	B	A	B	B	A	A	A	A	B	B
**Neurological level of injury**	C4	C4	C7	C4	C4	C4	C7	C4	C3	C6	C4	C7	C7	C6
**Implants**	Metronic - 2x Intellis IPG<br>Medtronic - 2x SureScan Specify 5.6.5			Metronic - 2x Intellis IPG<br>Medtronic - 2x SureScan Specify 5.6.5			ONWARD Medical - ARC-IM IPG<br>Medtronic - Specifiy SureScan 5.6.5				ONWARD Medical - ARC-IM IPG<br>ONWARD Medical - ARC-IM Thoracic Lead			ONWARD Medical - ARC-IM IPG<br>ONWARD Medical - ARC-IM Thoracic Lead
**Corresponding Figure**	Fig. 2<br>Fig. 4<br>Fig. 6a-e,g,h<br>ExtFig 1<br>ExtFig 3<br>ExtFig 6<br>ExtFig 9a-l<br>ExtFig 10d,e,h	Fig. 2<br>Fig. 4<br>Fig. 6a-e,g,h<br>ExtFig 1<br>ExtFig 3<br>ExtFig 6<br>ExtFig 9a-l<br>ExtFig 10d,e,h	Fig. 2<br>Fig. 4<br>Fig. 6a-e,g,h<br>ExtFig. 1<br>ExtFig. 3<br>ExtFig. 6<br>ExtFig. 9a-l,<br>ExtFig.10b,d,e,g,h	Fig. 2<br>Fig. 4<br>Fig. 6e,g,h<br>ExtFig. 1<br>ExtFig. 3<br>ExtFig. 6,<br>ExtFig. 10b,l,j	Fig. 2<br>Fig. 4<br>Fig. 6e,g,h<br>ExtFig. 1<br>ExtFig. 3<br>ExtFig. 6<br>ExtFig.10b,d,i	Fig. 2<br>Fig. 4<br>Fig. 6e,g,h<br>ExtFig. 1<br>ExtFig. 3<br>ExtFig. 6	Fig. 3<br>Fig. 4<br>Fig. 6<br>ExtFig. 4-6<br>ExtFig. 9<br>ExtFig. 10a-e,g,h	Fig. 3<br>Fig. 4<br>Fig. 6<br>ExtFig. 4-6<br>ExtFig.9,<br>ExtFig.10a, c-e,g,h,k	Fig. 3<br>Fig. 4<br>Fig. 6<br>ExtFig. 4-6,<br>ExtFig.9a-l,<br>ExtFig.10a-e,h,k	Fig. 3<br>Fig. 4<br>Fig. 6<br>ExtFig. 4-6,<br>ExtFig. 9,<br>ExtFig.10a-c,e,g,h,k	Fig. 3<br>Fig. 4<br>Fig. 6<br>ExtFig. 4-6,<br>ExtFig.9,<br>ExtFig.10a,c-e,g,h,k	Fig. 3<br>Fig. 4<br>Fig. 6<br>ExtFig. 4-6,<br>ExtFig. 9a-l,<br>ExtFig. 10a-e,g	Fig. 3<br>Fig. 4<br>Fig. 6<br>ExtFig.4-6<br>ExtFig.9a-k<br>ExtFig. 10a,c-e,g,h,k	Fig 6a-c,e-h<br>Ext Fig 8<br>ExtFig. 9<br>ExtFig.10a,d,f

Supplementary Data Table 2 | Patient demographics

Demographic and neurological status of participants. See **Supplementary Data 8** for additional details.

Trial Number	Short Study Name	Study Title	Sponsor	Investigational Site	Manufacturer	Principal Investigator	Study Type	Primary Endpoint Description	ClinicalTrial.gov	Ethics Reference Number	Competent Authorities Number	National Registry Number	Number of Implanted Patients as of 31.01.2024
1	STIMO-HEMO	Restoring Hemodynamic Stability Using Targeted Epidural Spinal Stimulation Following Spinal Cord Injury	CHUV	CHUV	MEDTRONIC	Jocelyne Bloch	Interventional, single group assignment	Investigate the preliminary safety of hemodynamic targeted epidural spinal stimulation (TESS) to modulate pressor responses and manage blood pressure instability in patients with chronic SCI located between C3 and T6 and who suffer from severe orthostatic hypotension (n=4).	NCT04994886	CER-VD 2021-00588	Swissmedic 10000882	SNCTP000004406	3
2	HEMO	Restoring Hemodynamic Stability Using Targeted Epidural Spinal Stimulation Following Spinal Cord Injury	University of Calgary	University of Calgary	MEDTRONIC	Aaron Phillips	Interventional, single group assignment	Investigate preliminary safety of hemodynamic targeted epidural spinal stimulation (TESS) to modulate pressor responses and manage blood pressure instability in patents with chronic SCI located between C3 and T6 who suffer from severe orthostatic hypotension (n=4)	NCT05044923	REB21-0027	not applicable	not applicable	3
3	HemON	Epidural Electrical Stimulation to Restore Hemodynamic Stability and Trunk Control in People With Spinal Cord Injury	EPFL	CHUV	ONWARD MEDTRONIC EPFL	Jocelyne Bloch	Interventional, single group assignment	Assess the safety of ARCIM Therapy at supporting the management of hemodynamic instability in participants with sub-acute or chronic spinal cord injury suffering from orthostatic hypotension.	NCT05111093	CER-VD 2021-D0025	Swissmedic 10000932	SNCTP000004706	7
4	HemON-NL	ARC Therapy to Restore Hemodynamic Stability and Trunk Control in People With Spinal Cord Injury	ONWARD	Sint Maartenskliniek	ONWARD	Ilse van Nes	Interventional, single group assignment	Assess the safety of ARC Therapy at supporting the management of hemodynamic instability in participants with sub-acute or chronic Spinal Cord Injury suffering from orthostatic hypotension.	NCT05941819	2023-16316 (METC)	NL83694.000.23 (CCMO)	not applicable	1

Supplementary Data Table 3 | Study information

Study information for each of the four clinical trials.

Event	Trial 1 NCT04994886	Trial 2 NCT05044923	Trial 3 NCT0511109 3	Trial 4 NCT05941819
**Number of implanted participants**	**3**	**3**	**7**	**1**
**Serious adverse events**	**3**	**4**	**3**	**0**
not related to device nor procedure	1	1	2	0
related to device	0	2	0	0
related to study procedure	2	1	1	0
**Non-serious adverse events**	**16**	**4**	**29**	**5**
not related to device nor procedure	3	3	16	3
related to device	5	1	4	2
related to study procedure	8	0	9	0

Supplementary Data Table 4 | Overview of (serious) adverse events

Serious and non-serious adverse events and relatedness to investigational device or study procedure reported in all four clinical trials: Trial 1 (STIMO-HEMO, NCT04994886), Trial 2 (HEMO, NCT05044923), Trial 3 (HemON, NCT05111093), and Trial 4 (HemON-NL, NCT05941819).

Reporting Period<br>Start: June 4, 2021<br>End: February 29, 2024	STIMO-HEMO			HEMO			HemON							HemON-NL
	P1	P2	P3	P4	P5	P6	P7	P8	P9	P10	P11	P12	P13	P14
Number of active months	20 Months	25 Months	14 Months	7 Months	13 Months	7 Months	22 Months	18 Months	18 Months	15 Months	10 Months	10 Months	3 Months	5 Months
**Number of Serious Adverse Device Effect (SADE)**	**0**	**2**	**0**	**1**	**1**	**1**	**0**	**0**	**1**	**0**	**0**	**0**	**0**	**0**
Related to device/related to procedure	0	0	0	1	0	1	0	0	0	0	0	0	0	0
Only related to study procedure	0	2	0	0	1	0	0	0	1	0	0	0	0	0
**Number of non-serious adverse device effect (ADE)**	**8**	**3**	**2**	**1**	**0**	**0**	**1**	**1**	**4**	**0**	**2**	**2**	**3**	**2**
Related to device/related to procedure	3	2	0	1	0	0	0	0	1	0	2	0	1	2
Only related to study procedure	5	1	2	0	0	0	1	1	3	0	0	2	2	0

Adverse device Effect (ADE) *(ISO 14155: 3.1): Adverse event possibly, probably, or causally related to the use of an investigational medical device. This definition includes adverse events resulting from insufficient or inadequate instructions for use, deployment, implantation, installation, or operation, or any malfunction of the investigational medical device. This definition includes any event resulting from use error or from intentional misuse of the investigational medical device. This includes comparator if the comparator is a medical device.*

Serious Adverse Device Effect (SADE) *(ISO14155)*: *Adverse device effect that has resulted in any of the consequences characteristic of a serious adverse event.*

Adverse events not related to device or procedure are not reported here.

When the (S)ADE is related to device, it has always extended to associated study procedure in this case. However, an adverse event might be related to study procedure while not being related to the device.

Supplementary Data Table 5 | Patient-by-patient safety data

Serious adverse device effects reported in all the participants of HemON and HemON-NL clinical trials.

STIMO-HEMO NCT04994886 Lausanne, Switzerland

Reporting Period
Start: June 4, 2021
End: January 31, 2024

Number	Subject	Short description	MedDra term	Device relation	Study relation	Severity	Status	Serious (Y/N)
1	P1	Left shoulder discomfort	Pain	Not related	Causal	Mild	RWOS	N
2	P1	Length of surgery		Causal	Causal	Moderate	RWOS	N
3	P1	Bruise on the left cheek	Bruise	Not related	Causal	Mild	RWOS	N
4	P1	Right shoulder discomfort	Pain	Not related	Causal	Moderate	RWOS	N
5	P1	Dizziness feeling	Dizziness	Not related	Possible	Mild	RWOS	N
6	P1	Discomfort in the trunk area	Discomfort	Probable	Causal	Mild	RWOS	N
7	P1	Knee and ankle bruise	Bruise	Not related	Possible	Moderate	RWOS	N
8	P2	Urinary infection following surgery	Urinary tract infection	Not related	Possible	Severe	RWOS	Y
9	P2	Urinary tract infection	Urinary tract infection	Not related	Possible	Severe	RWOS	Y
10	P1	Involuntary muscle contraction in finger	Muscle contractions involuntary	Causal	Causal	Mild	RWOS	N
11	P2	Urinary tract infection	Urinary tract infection	Not related	Possible	Moderate	RWOS	N
12	P3	Redness at incision site	Inflammation/implant site<br>Inflammation/incision site<br>Inflammation	Not related	Causal	Mild	RWOS	N
13	P3	Respiratory discomfort	Respiratory disorder	Not related	Possible	Moderate	RWOS	N
14	P2	Pain around abdominal suture site	Pain	Causal	Causal	Mild	Ongoing	N
15	P2	Involuntary muscle contraction in finger	Muscle contractions involuntary	Causal	Causal	Mild	Ongoing	N

Device relation: Not related, Possible, Probable, Causal
Study relation: Not related, Possible, Probable, Causal
Status: Resolved with sequelae (RWS), Resolved without sequelae (RWOS), Ongoing as of reporting end date
Severity: Mild, Moderate, Severe

Supplementary Data Table 6 | (Serious) Adverse Device Effects for STIMO-HEMO

HEMO

NCT05044923
Calgary,
Canada

Reporting period
Start: June 3, 2022
End: January 31, 2024

Number	Subject	Short description	MedDra term	Device relation	Study relation	Severity	Status	Serious (Y/N)
1	P4	Scar inflammation	Inflammation/incision site	Causal	Causal	Moderate	RWOS	N
2	P4	Infection 4 months after surgery and device explant	Infection	Possible	Possible	Severe	RWOS	Y
3	P5	Urinary tract infection following surgery	Urinary tract infection	Not related	Possible	Severe	RWOS	Y
4	P6	Scar inflammation and systemic infection and device explant	Infection	Causal	Causal	Severe	RWOS	Y

Device relation: Not related, Possible, Probable, Causal
Study relation: Not related, Possible, Probable, Causal
Status: Resolved with sequelae (RWS), Resolved without sequelae (RWOS), Ongoing as of reporting end date
Severity: Mild, Moderate, Severe

Supplementary Data Table 7 | (Serious) Adverse Device Effects for HEMO

HemON NCT05111093 Lausanne, Switzerland — Reporting Period Start: March 18, 2022 End: February 29, 2024

Number	Subject	Short description	MedDra term	Device relation	Study relation	Severity	Status	Serious (Y/N)
1	P8	Neuropathic pain at puncture site during blood sampling, accompanied by insomnia	Pain	Not related	Possible	Mild	RWOS	N
2	P9	Transient unexpected sensation due to overstimulation	Discomfort	Causal	Causal	Mild	RWOS	N
3	P9	Blisters around stitches	Blister	Not related	Causal	Mild	RWOS	N
4	P9	Urgency autonomic dysreflexia	Autonomic Dysreflexia	Not related	Probable	Severe	RWOS	Y
5	P9	Supine hypertension after tilt and transient headache episode	Hypertension	Not related	Causal	Moderate	RWOS	N
6	P9	Neuropathic pain at puncture site during blood sampling	Pain	Not related	Causal	Mild	RWOS	N
7	P7	Skin irritation	Skin irritation	Not related	Causal	Mild	RWOS	N
8	P11	Pain in abdominal region and constipation	Implant site pain	Possible	Probable	Moderate	RWOS	N
9	P12	Malleolus fracture	Fracture	Not related	Probable	Moderate	RWOS	N
10	P12	Bruises following surgery	Bruise	Not related	Causal	Mild	RWOS	N
11	P11	High pressure accompanied by AD-like symptoms	Autonomic Dysreflexia	Causal	Causal	Mild	RWOS	N
12	P13	Transient unexpected sensation due to stimulation	Discomfort	Causal	Causal	Mild	RWOS	N
13	P13	Suture loosening	Suture rupture	Not related	Causal	Moderate	RWOS	N
14	P13	Urinary Tract Infection accompanied by AD-like symptoms	Urinary Tract Infection/Autonomic Dysreflexia	Not related	Possible	Moderate	RWOS	N

HemON-NL NCT05941819 Nijmegen, Netherlands — Reporting Period Start: September 3, 2023 End: February 29, 2024

Number	Subject	Short description	MedDra term	Device relation	Study relation	Severity	Status	Serious (Y/N)
1	P14	Fever	Fever	Possible	Possible	Mild	RWOS	N
2	P14	Temperature elevation	Body temperature increased	Probable	Probable	Mild	RWOS	N

Device relation: Not related, Possible, Probable, Causal
Study relation: Not related, Possible, Probable, Causal
Status: Resolved with sequelae (RWS), Resolved without sequelae (RWOS), Ongoing as of reporting end date
Severity: Mild, Moderate, Severe

Supplementary Data Table 8 | (Serious) Adverse Device Effects for HemON and HemON-NL
