# Supplementary material for: An implantable system to restore hemodynamic stability after spinal cord injury
Source: Nat Med. 2025 Sep 17;31(9):2946–57. doi: 10.1038/s41591-025-03614-w (PMC12443590; doi:10.1038/s41591-025-03614-w)
Supplement: Supplementary file 2 — Reporting Summary [file 41591_2025_3614_MOESM2_ESM.pdf]

nature portfolio

Corresponding author(s): Gregoire Courtine
Jocelyne Bloch
Aaron Phillips

Last updated by author(s): Jan 21, 2025

Reporting Summary

Nature Portfolio wishes to improve the reproducibility of the work that we publish. This form provides structure for consistency and transparency in reporting. For further information on Nature Portfolio policies, see our Editorial Policies and the Editorial Policy Checklist.

Statistics

For all statistical analyses, confirm that the following items are present in the figure legend, table legend, main text, or Methods section.

n/a	Confirmed	
☐	☒	The exact sample size (*n*) for each experimental group/condition, given as a discrete number and unit of measurement
☐	☒	A statement on whether measurements were taken from distinct samples or whether the same sample was measured repeatedly
☐	☒	The statistical test(s) used AND whether they are one- or two-sided<br>*Only common tests should be described solely by name; describe more complex techniques in the Methods section.*
☐	☒	A description of all covariates tested
☐	☒	A description of any assumptions or corrections, such as tests of normality and adjustment for multiple comparisons
☐	☒	A full description of the statistical parameters including central tendency (e.g. means) or other basic estimates (e.g. regression coefficient) AND variation (e.g. standard deviation) or associated estimates of uncertainty (e.g. confidence intervals)
☐	☒	For null hypothesis testing, the test statistic (e.g. *F*, *t*, *r*) with confidence intervals, effect sizes, degrees of freedom and *P* value noted<br>*Give P values as exact values whenever suitable.*
☒	☐	For Bayesian analysis, information on the choice of priors and Markov chain Monte Carlo settings
☒	☐	For hierarchical and complex designs, identification of the appropriate level for tests and full reporting of outcomes
☒	☐	Estimates of effect sizes (e.g. Cohen's *d*, Pearson's *r*), indicating how they were calculated

Our web collection on statistics for biologists contains articles on many of the points above.

Software and code

Policy information about availability of computer code

Data collection	All softwares and software versions used to acquire data: G-Drive Plus software up to v3.2 suite by EPFL, EMG Works 4.7.8 and Delsys Trigno Software, LabChart version 7 or later, IOMAX from Cadwell Industries or ISIS Xpress from Inomed Medizintechnik, Finapres Nova with up to date device firmware, 3T MRI with either a Magnetom PrismaFit (Siemens Healthineers, Eriangen, Germany), Discovery MR750 (GE Healthcare, Waukesha, WI, USA), or Ingenia Omega (Philips Healthcare, Best, The Netherlands).<br><br>All data collection methods, procedures and devices are detailed in the main text of the manuscript, and in the relevant methods sections.
Data analysis	All softwares and corresponding versions used to analyze data:<br>- Matlab v2018a and later by Mathworks<br>- RStudio 2023.12.0+369 and later, R version 4.3.2 and later<br>- Python version 3.2 and later<br>- Sim4Life version 7 and later<br>- Illustrations were generated using Adobe illustrator 2022, R (version 4.3.2 and later), Blender version 4.0 and later<br><br>Additional details are provided in the methods section.

For manuscripts utilizing custom algorithms or software that are central to the research but not yet described in published literature, software must be made available to editors and reviewers. We strongly encourage code deposition in a community repository (e.g. GitHub). See the Nature Portfolio guidelines for submitting code & software for further information.

Data

Policy information about availability of data

All manuscripts must include a data availability statement. This statement should provide the following information, where applicable:

- Accession codes, unique identifiers, or web links for publicly available datasets
- A description of any restrictions on data availability
- For clinical datasets or third party data, please ensure that the statement adheres to our policy

All data supporting the findings of this study are provided in the Supplementary Information (Supplementary Data 1-8) and in the following data depository 10.5281/zenodo.14714233. Access to additional raw data beyond the supplementary files may be subject to reasonable restrictions due to data privacy regulations and institutional policies. Requests for additional data should be directed to the corresponding authors and will be reviewed based on the purpose of the request and compliance with ethical and data-sharing guidelines.
Software routines developed for the data analysis will be made available upon reasonable request to the corresponding authors at gregoire.courtine@epfl.ch, jocelyne.bloch@chuv.ch, or aaron.phillips@ucalgary.ca.

Research involving human participants, their data, or biological material

Policy information about studies with human participants or human data. See also policy information about sex, gender (identity/presentation), and sexual orientation and race, ethnicity and racism.

Reporting on sex and gender

Information on sex (female/male) was determined based on self-reporting and is provided in Data Table 1 (14 participants). Gender information was not explicitly collected during the study.

Reporting on race, ethnicity, or other socially relevant groupings

The categorization of individuals into different ethnic groups is based on the concept of ethnicity that pertains to an individual's cultural and social identity, encompassing shared traditions, language, heritage, and historical connections to particular social or cultural groups. Race, ethnicity, or other socially relevant grouping information was not explicitly collected or analyzed during this trial.

Population characteristics

Patients fulfilling eligibility criteria and willing to provide informed consent were enrolled. Informed consent was obtained for all participants. Age, weight, height, sex, and injury level were collected as demographic information (refer to Data Table 1 and Supplementary Data 8 for details).

The participants received no explicit compensation for the study. All travel expenses to and from the hospital were covered by the sponsor of the study, and the necessary logistics were put in place for travel and on-site accommodation during their stay. If a caregiver was required for support in daily life, travel and on-site accommodation for the caregiver was also provided.

Study population characteristics are described by each study :

STIMO-HEMO (NCT 04994886; Lausanne, Switzerland)
The study involved 3 individuals with spinal cord injuries and all with neurological severity AIS A (3 males, 0 females, 31.3 +/- 2.3 years of age). Sex and gender were self-reported. Informed consent was obtained for all participants. The participants received no compensation for the study.

Patients fulfilling the following criteria were eligible:
• Age 18 to 70 years old
• Able to undergo the informed consent/assent process
• Radiologically confirmed spinal cord injury
• Spinal cord injury between C3 and T6
• Classified with AIS A or B Spinal cord injury
• Stable medical, physical and psychological condition as considered by Investigators
• Greater than 1 year since initial injury and at least 6 months from any required spinal instrumentation
• Confirmed orthostatic hypotension and autonomic dysreflexia
• Willing to attend all scheduled appointments

Patients with the presence of any of the following exclusion criteria were ineligible:
• Patients in an emergency situation
• Diseases and conditions that would increase the morbidity and mortality of spinal cord injury surgery
• The inability to withhold antiplatelet/anticoagulation agents perioperatively
• History of myocardial infarction or cerebrovascular event within the past 6 months
• Other conditions that would make the subject unable to participate in testing in the judgment of the investigators
• Current and anticipated need for opioid pain medications or pain that would prevent full participation in the rehabilitation program in the judgement of the investigators
• Clinically significant mental illness in the judgment of the investigators
• Botulinum toxin injections in the previous 6 months
• Presence of significant pressure ulcers
• Recurrent urinary tract infection refractory to antibiotics
• Current pregnancy
• Current breast feeding
• Known or suspected drug or alcohol abuse
• Unhealed spinal fractures

• Presence of indwelling baclofen or insulin pump

HEMO (NCT05044923; Calgary, Canada)
The study included 3 individuals with spinal cord injuries and all with neurological severity AIS A and AIS B (2 males, 1 female, 34.7 +/- 9.3 years of age). Sex and gender were self-reported. The participants received no compensation for the study.

Patients fulfilling the following criteria were eligible:
• Age 18 to 70 years old
• Able to undergo the informed consent/assent process
• Radiologically confirmed spinal cord injury
• Spinal cord injury between C3 and T6
• Classified with AIS A or B Spinal cord injury
• Stable medical, physical and psychological condition as considered by Investigators
• Greater than 1 year since initial injury and at least 6 months from any required spinal instrumentation
• Confirmed orthostatic hypotension and autonomic dysreflexia
• Willing to attend all scheduled appointments

Patients with the presence of any of the following exclusion criteria were ineligible:
• Diseases and conditions that would increase the morbidity and mortality of spinal cord injury surgery
• The inability to withhold antiplatelet/anticoagulation agents perioperatively
• History of myocardial infarction or cerebrovascular event
• Other conditions that would make the subject unable to participate in testing in the judgment of the investigators
• Current and anticipated need for opioid pain medications or pain that would prevent full participation in the trial in the judgement of the investigators
• Current clinical diagnosis of mental illness
• Clinically significant cognitive impairment as assessed using the MoCA-Blind
• Current substance or alcohol abuse as assessed using the CAGE questionnaire
• Botulinum toxin injections in the previous 6 months
• Presence of significant pressure ulcers
• Recurrent urinary tract infection refractory to antibiotics
• Current pregnancy
• Current breast feeding
• Unhealed spinal fractures
• Presence of indwelling baclofen or insulin pump

HemON (NCT05111093; Lausanne, Switzerland)
The study included 7 individuals with spinal cord injuries and all with neurological severity AIS A and AIS B (3 males, 4 females, 42.9 +/- 18.7 years of age). Sex and gender were self-reported. Informed consent was obtained for all participants. The participants received no compensation for the study.

Patients fulfilling the following criteria were eligible:
• 18 years of age or older
• Must provide and sign the Informed Consent prior to any related procedures
• Spinal cord injury lesion level between C3 and T6 (inclusive)
• SCI ≥ 1month
• Confirmed orthostatic hypotension
• Stable medical, physical and psychological condition as considered by the investigators
• Able to understand and interact with the study team in Fr English
• Agrees to comply in good faith with all conditions of the studu to attend all scheduled appointments

Patients with the presence of any of the following exclusion criteria were ineligible:
• SCI related to a neurodegenerative disease
• Diseases and conditions that would increase the morbid mortality of spinal cord injury surgery
• The inability to withhold antiplatelet/anticoagulation perioperatively
• History of myocardial infarction or cerebrovascular even the past 6 months
• Other conditions that would make the subject unable to par in testing in the judgement of the investigators
• Clinically significant mental illness in the judgement investigators
• Botulinum toxin non-vesical and vesical injections in the past 3 months before the enrollment
• Presence of significant pressure ulcers
• Recurrent urinary tract infection refractory to antibiotics
• Presence of indwelling baclofen or insulin pump
• Women who are pregnant (pregnancy test obligatory for of childbearing potential) or breast feeding,
• Lack of safe contraception for women of childbearing capacity
• Intention to become pregnant during the course of the study
• Other clinically significant concomitant disease states (e.g failure, hepatic dysfunction, cardiovascular disease, etc.),
• Inability to follow the procedures of the study, e.g. due to la problems, psychological disorders, or dementia of the part
• Participation in another study with investigational drug wit 30 days preceding and during the present study,
• Enrollment of the investigator’s, his/her family, or former employees and other dependent persons.

HemON-NL (NCT05941819; Nijmegen, The Netherlands)
The study involved 1 individual with spinal cord injuries and all with neurological severity AIS B (1 male, 31 years of age). Sex and gender were self-reported. Informed consent was obtained for all participants. The participants received no compensation for the study.

Patients fulfilling the following criteria were eligible:

• 18 years of age or older
• Must provide and sign the Informed Consent prior to any study-related procedures
• Traumatic Spinal Cord Injury
• Spinal cord injury lesion level between C3 and T6 (inclusive)
• AIS- A, B, C or D
• SCI ≥ 1month
• Confirmed orthostatic hypotension
• Stable medical, physical and psychological condition as considered by the investigators
• Able to understand and interact with the study team in Dutch or English
• Agrees to comply in good faith with all conditions of the study and to attend all scheduled appointments
• In case participants need continuous support from a personal caregiver in daily life, then the presence of their caregiver during the visits to the study site is needed, including independent transport (not dependent on a cab)

Patients with the presence of any of the following exclusion criteria were ineligible:
• Diseases and conditions that would increase the morbidity and mortality of spinal cord injury surgery
• Diseases and conditions that would require regular MRI
• The inability to perform an MRI due to metal, magnetic or electrical device in the body (e.g. oral implant with magnet, metal splinter, neurostimulator, artificial heart valve, clips, stents...) as assessed by the MRI form of Sint Maartenskliniek
• The inability to withhold antiplatelet/anticoagulation agents perioperatively
• History of myocardial infarction or cerebrovascular event within the past 6 months
• Other conditions that would make the subject unable to participate in testing in the judgement of the investigators
• Clinically significant mental illness in the judgement of the investigators
• Botulinum toxin non-vesical and vesical injections in the previous 3 months before the enrollment
• Presence of significant pressure ulcers
• Recurrent urinary tract infection refractory to antibiotics
• Presence of indwelling baclofen (e.g. intrathecal baclofen pump) or insulin pump
• Women who are pregnant (pregnancy test obligatory for woman of childbearing potential) or breast feeding
• Lack of safe contraception for women of childbearing capacity
• Intention to become pregnant during the course of the study,
• Other clinically significant concomitant disease states (e.g., renal failure, hepatic dysfunction, cardiovascular disease, etc.)
• Inability to follow the procedures of the study, e.g. due to language problems, psychological disorders, or dementia of the participant
• Participation in another study with an investigational drug within the 30 days preceding and during the present study
• Enrollment of the investigator, his/her family members, employees, and other dependent persons

Recruitment

Participants could only be recruited after obtaining approval from the competent authorities and ethical committees (see Ethics oversight below).

Investigators were responsible for screening patients for enrollment in the study. Participants may be recruited within the list of patients commonly followed by the research and clinical department at the hospital or may be referred to the principal investigator via their networks from other primary care facilities and/or rehabilitation institutions. A first contact is then established between the principal investigator and eligible patients. During this first visit, the study will be presented to the eligible patients via a printed information sheet. The participants receive complete oral and written information about the investigation using non-technical language and were given 72 hours to decide whether they would like to participate. No direct financial incentive was offered to the participants although fees related to travel expenses and lodging are covered/ reimbursed by the study.
We screen and enroll participants on a rolling basis. This strategy may introduce self-selection bias by favoring individuals who are more proactive or have greater time availability.

Ethics oversight

The STIMO-HEMO study was approved by the Swiss ethical authorities (Ethics reference number: CER-VD 2021-00588) and is conducted following the Declaration of Helsinki (clinicaltrials.gov ID: NCT 04994886). The sponsor submitted an investigation to Swissmedic and obtained regulatory approval before the start of the study (Competent authorities' number: Swissmedic 10000882).

The HEMO study was approved by the Canadian ethical authorities (Ethics reference number: REB21-0027) and is conducted following the Declaration of Helsinki (clinicaltrials.gov ID: NCT05044923).

The HemON study was approved by the Swiss ethical authorities (Ethics reference number: CER-VD 2021-D0025) and is conducted following the Declaration of Helsinki (clinicaltrials.gov ID: NCT05111093). The sponsor submitted an investigation to Swissmedic and obtained regulatory approval before the start of the study (Swissmedic 10000932).

The HemON-NL study was approved by the Dutch ethical authorities (Ethics reference number: METC 2023-16316) and is conducted following the Declaration of Helsinki (clinicaltrials.gov ID: NCT NCT05941819). The sponsor submitted an investigation to the Central Committee on Research Involving Human Subjects, and obtained regulatory approval before the start of the study (CCMO NL83694.000.23).

Additional details regarding study information can be found in Supplementary Data Table 1.

Note that full information on the approval of the study protocol must also be provided in the manuscript.

Field-specific reporting

Please select the one below that is the best fit for your research. If you are not sure, read the appropriate sections before making your selection.

☒ Life sciences ☐ Behavioural & social sciences ☐ Ecological, evolutionary & environmental sciences

For a reference copy of the document with all sections, see nature.com/documents/nr-reporting-summary-flat.pdf

Life sciences study design

All studies must disclose on these points even when the disclosure is negative.

Sample size

All four studies have observational, primary feasibility and safety endpoints. Hence, no sample size is required for the primary endpoints. Primary and secondary endpoints aim to evaluate the feasibility and safety of the procedure, investigational devices, and the methodologies used. Safety, efficacy and exploratory outcomes will be assessed for all patients enrolled in the study and who received the implant.

STIMO-HEMO: Up to four participants were anticipated to be implanted. Of the four enrolled thus far, 3 met the inclusion and exclusion criteria and were implanted with the investigational device. The outcomes in this study are reported on the 3 implanted participants.
HEMO: Up to four participants were anticipated to be implanted. Of the four enrolled thus far, 3 met the inclusion and exclusion criteria and were implanted with the investigational device. The outcomes in this study are reported on the 3 implanted participants.
HemON: Up to 12 participants are anticipated to be implanted. Of the 10 enrolled thus far, 7 met the inclusion and exclusion criteria and were implanted with the investigational device. The outcomes in this study are reported on the 7 implanted participants.
HemON-NL: Up to 8 participants are anticipated to be implanted. Of the 4 enrolled thus far, 2 met the inclusion and exclusion criteria and 1 was implanted with the investigational device. The outcomes in this study are reported on the 1 implant partcipant thus far.

Data exclusions

Participant exclusions: Participants screened but not enrolled and implanted with the investigational devices were excluded from any analysis.

Data from continuous blood pressure monitoring was excluded if technical issues with the Finometer were observed including loss of the plethysmographic signal, incorrect calibration as measured by comparing continuous measuring to standard measurements from a brachial cuff, spasms, or due to study protocol deviations or adverse events.

Replication

Official blood pressure assessments were repeated once every time point (refer Extended Data Figure 1, 3, 6 and 7 for clinical trial timelines). Official blood pressure assessments were conducted according to the protocol, with repeats performed only in cases of technical issues or other specific complications.

Quality of life related questionnaires and surveys are queried and collected monthly after the configuration phase of each trial. All other blood pressure assessments were repeated at least 3 times to ensure safety and efficacy of the stimulation.

Randomization

Randomization for human participants was not sought in the present study. Each participant served as their own control (stimulation off vs. on conditions; evaluations at different points over time throughout the rehabilitation training period).

Blinding

For all clinical trials, investigators were not blinded. Their expertise was required to optimize the intervention and to apply the intervention during evaluations. Furthermore, the effects of the intervention were obvious, acutely producing changes in the blood pressure or muscle activity with and without stimulation.

Reporting for specific materials, systems and methods

We require information from authors about some types of materials, experimental systems and methods used in many studies. Here, indicate whether each material, system or method listed is relevant to your study. If you are not sure if a list item applies to your research, read the appropriate section before selecting a response.

Materials & experimental systems

n/a	Involved in the study	
☒	☐	Antibodies
☒	☐	Eukaryotic cell lines
☒	☐	Palaeontology and archaeology
☒	☐	Animals and other organisms
☐	☒	Clinical data
☒	☐	Dual use research of concern
☒	☐	Plants

Methods

n/a	Involved in the study	
☒	☐	ChIP-seq
☒	☐	Flow cytometry
☐	☒	MRI-based neuroimaging

Clinical data

Policy information about clinical studies

All manuscripts should comply with the ICMJE guidelines for publication of clinical research and a completed CONSORT checklist must be included with all submissions.

Clinical trial registration	STIMO-HEMO Clinicaltrials.gov ID: NCT04994886, SNCTP000004406<br>HEMO Clinicaltrials.gov ID: NCT05044923<br>HemON Clinicaltrials.gov ID: NCT05111093, SNCTP000004706<br>HemON-NL Clinicaltrials.gov ID: NCT05941819
Study protocol	STIMO-HEMO: https://clinicaltrials.gov/study/NCT04994886<br>HEMO: https://clinicaltrials.gov/study/NCT05044923<br>HemON: https://clinicaltrials.gov/study/NCT05111093<br>HemON-NL: https://clinicaltrials.gov/study/NCT05941819
Data collection	STIMO-HEMO: Investigational site is at CHUV (Lausanne, Switzerland); data was collected between June 2021 and January 2024 in three participants.<br>HEMO: Investigational site is at the University of Calgary (Calgary, Canada); data was collected between April 2022 and January 2024 in three participants<br>HemON: Investigational site is at the CHUV (Lausanne, Switzerland); data was collected between April 2022 and February 2024 in 7 participants.<br>HemON-NL: Investigational site is at the Sint Maartenskliniie (Nijmegen, The Netherlands); data was collected between September 2023 and February 2024 in 1 participant.

Outcomes

STIMO-HEMO (NCT04994886; Lausanne, Switzerland):
Primary outcome: The safety measure will report on the occurrence of Adverse Events and Serious Adverse Events that are deemed related or possibly related to the study procedure or to the study investigational system, from implant surgery until the end of study. The safety population will account for all patients enrolled in the study and who received the implant. The implant procedure is similar to the procedure for a chronic pain implant. However, the study procedures combine two implants and additional testing measures. Therefore, it is expected that the number of patients with safety outcomes will be slightly higher than the publicly reported safety of chronic pain implants.

Secondary Outcome: The secondary outcome will report on the efficacy measure and will quantify the immediate ability of TESS to trigger pressor response and to manage blood pressure instability during orthostatic challenges. The efficacy population will account for all implanted patients who have completed the study. It is expected that patients who have received hemodynamic TESS will exhibit improvements in their outcome measures of cardiovascular health.

HEMO (NCT05044923; Calgary, Canada)
Primary outcome: The safety measure will report on the occurrence of Adverse Events and Serious Adverse Events that are deemed related or possibly related to the study procedure or to the study investigational system, from implant surgery until the end of study. The safety population will account for all enrolled patients eligible for implant surgery. Patients enrolled in the study but withdrawn before implant will not account for the safety population. The implant procedure is similar to the procedure for a chronic pain implant. However, the study procedures combine two implants and additional testing measures. Therefore, it is expected that the number of patients with safety outcomes will be slightly higher than the publicly reported safety of chronic pain implants.

Secondary Outcome: The secondary outcome will report on the efficacy measure and will quantify the immediate ability of TESS to trigger pressor response and to manage blood pressure instability during orthostatic challenges. The population will account for all implanted patients who have completed the study. It is expected that patients who have received hemodynamic TESS will exhibit improvements in their outcome measures of cardiovascular health.

HemON (NCT05111093; Lausanne, Switzerland):

Primary outcome: Occurrence of serious adverse events and adverse events that are deemed related or possibly related to the study procedure or to the ARCIM Investigational System, from implantation up to the end of the study.
Secondary outcomes with clinical impact: 1) Preliminary efficacy of ARCIM Therapy in supporting management of hemodynamic instability will be assessed with orthostatic head-up tilt tests measured by continuous blood pressure monitoring, a seated blood pressure measurement, a cerebral blood flow assessment, a post-prandial assessment, and with the Mapping of Rehab Training (MART) assessments filled by therapists during rehabilitation sessions. 2) Effect of ARCIM Therapy on trunk control and mobility will be assessed with the Wheelchair Performance test, the Trunk Control Test, trunk stability measurements, FIST-SCI test and CT scans. 3) Effect of ARCIM Therapy on muscle tone normalization will be evaluated with spasticity questionnaires filled by the participants and via the Modified Ashworth Scale assessment (MAS). 4) Effect of ARCIM Therapy on daily life performance will be assessed with the SCIM III score (Spinal Cord Independence Measure) and with the COPM (Canadian Occupational Performance Measure).
Secondary outcomes with technical impact: 1) Evaluation of the independent use of ARCIM Investigational System will be assessed with a stimulation usage log and weekly questionnaires filled by the participants. 2) Evaluation of the robustness of ARCIM Investigational System will be assessed based on the occurrence of Device Deficiencies, and by analysing the automatic logs recorded by the devices. 3) Evaluation of the usability and clinical procedures of the ARCIM Investigational System by the different users will be assessed with a surgical checklist and usability testing to evaluate different scenarios and questionnaires to perform with the system. 4) Evaluation of ARCIM Investigational System requirements and specification will be assessed with lead design tests, biomimetic stimulation tests, and ramping tests of the ARCIM IPG during optimization sessions.

HemON-NL (NCT05941819; Nijmegen, The Netherlands):

Primary outcome: Occurrence of serious adverse events and adverse events that are deemed related or possibly related to the study

procedure or to the ARCIM Thoracic System, from implantation up to the end of the study.
Secondary outcomes with clinical impact: 1) Preliminary effectiveness of ARC Therapy on supporting management of orthostatic hypotension will be assessed via orthostatic head-up tilt tests and via the Mapping of Rehab Training (MART) forms filled by therapists during rehabilitation sessions. 2) Preliminary effectiveness of ARC Therapy on supporting average blood pressure management will be assessed via continuous blood pressure monitoring using a wearable device. 3) Preliminary effectiveness of ARC Therapy on supporting trunk control will be assessed with the Trunk Control Test (TCT), trunk stability measurements, wheelchair performance test, the Function In Sitting Test (FIST SCI) and CT scans. 4) Preliminary effectiveness of ARC Therapy on supporting spasticity will be evaluated with spasticity questionnaires filled by the participants and via the Modified Ashworth Scale (MAS). 5) Preliminary effectiveness of ARC Therapy on supporting daily life performance will be assessed with the Spinal Cord Independence Measure (SCIM III), with the Canadian Occupational Performance Measure (COPM) and with a daily life activity monitoring assessment.
Secondary outcomes with technical impact: 1) Evaluation of the independent use of ARCIM Thoracic System will be assessed with a stimulation usage log and weekly questionnaires filled by the participants. 2) Evaluation of the robustness of the ARCIM Thoracic System will be assessed based on the occurrence of Device Deficiencies, and by analyzing the automatic logs recorded by the devices. 3) Evaluation of the usability and clinical procedures of the ARCIM Thoracic System by the different users will be assessed with a surgical checklist and questionnaires to be filled about the system.

Plants

Seed stocks	n/a
Novel plant genotypes	n/a
Authentication	n/a

Magnetic resonance imaging

Experimental design

Design type	Participants were positioned supine with arms at their sides.
Design specifications	Resting state structural MRI. The scan time was less than 90 minutes for each participant depending on the sequence parameters (see below).
Behavioral performance measures	The participant did not perform any behavioral experiment while being scanned (no fMRI was acquired on the human participant). The MRI scan was an anatomical structural volume imaging with the participant at rest.

Acquisition

Imaging type(s)	Structural MRI
Field strength	3 Tesla
Sequence & imaging parameters	STIMO-HEMO and HemON<br>MRI was performed on a Magnetom PrismaFit (Siemens Healthineers, Erlangen, Germany). The standard MRI protocol comprised the following four pulse sequences: a) 2D sagittal T2-weighted turbo spin-echo (repetition time (TR), 3080 msec; echo time (TE), 98 msec; voxel size, 0.6×0.6×3 mm3); b) T2-weighted SPACE (Sampling Perfection with Application-optimized Contrasts using different flip angle Evolution) sequence (TR, 1500 msec; TE, 135 msec; interpolated voxel size, 0.4 x 0.4 x 0.8 mm3); c) 3D axial T2-weighted SPACE with ZOOMit (dynamic excitation pulses to achieve selective/zoomed field-of-view) software (TR, 2500 msec; TE, 106 msec; interpolated voxel size, 0.3×0.3×0.5 mm3); and d) 3D coronal T2-weighted TrueFISP (True Fast Imaging with Steady state Precession) (TR, 6.04 msec; TE, 3.02 msec; interpolated voxel size, 0.3×0.3×0.6 mm3).<br><br>HEMO<br>MRI was performed on a Discovery MR750 (GE Healthcare, Waukesha, WI, USA) with an 8-channel cervical thoracic lumbar coil. The following two sequences were performed: a) 3D coronal T2-weighted FIESTA-C (Fast Imaging Employing Steady-state Acquisition) (TR, 7.65 msec; TE, 3.48 msec; voxel size, 0.4×0.4×0.4 mm3); and b) 3D sagittal T2-weighted CUBE sequence (TR, 2000 msec; TE, 90 msec; interpolated voxel size, 0.8×1.25×0.8 mm3).<br><br>HemON-NL<br>MRI was performed on an Ingenia Omega (Philips Healthcare, Best, The Netherlands) with a 32-channel coil. The protocol comprised the following two sequences: a) sagittal T2-weighted FFE (Fast Field Echo) (TR, 3.34 msec; TE, 8.1 msec; interpolated voxel size, 0.75×0.75×3 mm3); and b) coronal Balanced FFE (TR, 7.0 msec; TE, 3.5 msec; interpolated voxel size, 0.75×0.75×3 mm3).
Area of acquisition	Thoraco-lumbar spinal cord

Diffusion MRI ☐ Used ☒ Not used

Preprocessing

Preprocessing software	MRI image acquisition did not require a preprocessing software. No preprocessing software was used.
Normalization	MRI image acquisition did not require normalization. Normalization was not used.
Normalization template	MRI image acquisition did not require a normalization template. Normalization template was not used.
Noise and artifact removal	Shim boxes were applied to correct for magnetic field inhomogeneities
Volume censoring	MRI image acquisition did not require volume sensoring. Volume censoring was not used.

Statistical modeling & inference

Model type and settings	n/a
Effect(s) tested	n/a

Specify type of analysis: ☐ Whole brain ☐ ROI-based ☐ Both

Statistic type for inference<br>(See Eklund et al. 2016)	n/a
Correction	n/a

Models & analysis

n/a	Involved in the study
☒	☐ Functional and/or effective connectivity
☒	☐ Graph analysis
☒	☐ Multivariate modeling or predictive analysis
